# Regulation of Immunity in Clear Cell Renal Carcinoma: Role of PD-1, PD-L1, and PD-L2

**DOI:** 10.3390/cimb43020076

**Published:** 2021-09-06

**Authors:** Liudmila Spirina, Zahar Yurmazov, Evgeny Usynin, Irina Kondakova, Ekaterine Ladutko, Evgeny Choynzonov

**Affiliations:** 1 Cancer Research Institute, Tomsk National Research Medical Center, 634050 Tomsk, Russia; pzahar76@gmail.com (Z.Y.); gusi70@list.ru (E.U.); kondakova@oncology.tomsk.ru (I.K.); info@oncology.tomsk.ru (E.C.); 2 Siberian State Medical University, 634050 Tomsk, Russia; ladutkoekaterina@yandex.ru (E.L.);

**Keywords:** kidney cancers, PD-1, PD-L1, PD-L2, primary tumor, metastasis, molecular factors

## Abstract

Regulation of immunity is a unique oncogenic mechanism that differs in different cancers. VHL deficient clear cell renal cell carcinomas (ccRCC) trigger the immune response resulting in cancer progression. This study aimed to investigate PD-1, PD-L1, and PD-L2 expression in ccRCC primary cancers and metastatic tissues associated with the p-VHL content, transcriptional, and growth factors expression. Methods: A total of 62 patients with RCC were enrolled in the study. Investigation of mRNA level was performed by PCR in real-time. Western blotting analysis was used for detecting the p-VHL protein content in tissues. Results: The PD-L2 prevalence in metastatic cancers is crucial in tumor progression. The VHL expression and p-VHL content determined the aggressive cancer behavior and elevated in disseminated tumors. The cancer dissemination was accompanied by an increase in both mRNA and VHL content. Conclusion: We present a new instrument targeting pathologies with p-VHL/HIF altered function that impact the PD-L2 expression through the change in transcriptional, growth factors, and AKT/mTOR modulation.

## 1. Introduction

Tumor immunogenicity is one of the urgent problems in modern oncology. Receptor PD-1 (Programmed cell death 1; CD279) plays a role in the cellular differentiation of immune cells [1]. Under physiological conditions, when PD-1 interacts with its ligands (PD-L1, PD-L2), an inhibitory signal is transmitted that prevents the development of an excessive immune response, triggering the processes of apoptosis of cytotoxic lymphocytes [2].

The recent analysis highlights the VHL impact on the biological processes, including metabolism, immune regulation, apoptosis, and cell movement. Its overexpression promotes immune system activation and sensitivity to interferon therapy [3]. The studies showed that PD-L1 expression levels positively correlate with VHL mutation and HIF-2α expression. VHL mutations positively correlate with PD-L1 expression in ccRCC and may influence ccRCC anti-PD-L1/PD-1 immunotherapy [4].

In the presented works, the relationship of the positive expression of PD-L1 with the sporadic and hereditary ccRCC aggressiveness is currently noted [5]. It was revealed that an association between the VHL biallelic inactivation and the PD-L1 increased expression [4]. In addition, there is evidence that if protein E3 of ubiquitin ligase is functional, an increase in the PD-L1/PD-1 will be observed [6]. Unlike PD-L1, PD-L2 has a less pronounced association with disease prognosis [7].

Currently, the prognostic role of the PD-L1 level with the cancer aggressiveness and the unfavorable outcome development is prominent [8]. Its function is currently not clearly defined. However, blocking antitumor immunity and inhibition of cytotoxic T cells and NK cells is possible, among other things, by increasing the expression of PD-L2 [9]. However, there is evidence of a greater affinity of this ligand for PD-1 receptors of immunocompetent cells [10]. It is known that the expression of PD-1, PD-L1, and PD-L2 differs in primary tumor tissues and metastases [11].

The changes in AKT/mTOR and MAPK signaling cascades in the ccRCC can influence tumorigenesis [12,13]. In addition, the expression of p-AKT is closely associated with the expression of ligands PD-L1 and PD-L2 in tumor cells [14]. The tumor withdrawal from immune surveillance occurs due to the PD-L1 and PD-L2 production by tumor cells is associated with the hypoxia initiation and the HIF-1 protein level [15,16]. In particular, for kidney cancer, a PD-L1 association with a HIF-2α high level in the presence of lymphocytic infiltration has been shown [17,18,19]. The study aimed to investigate PD-1, PD-L1, and PD-L2. Expression in ccRCC primary cancers and metastatic tissues associated with the p-VHL content, transcriptional, and growth factors expression.

## 2. Materials and Methods

A total of 62 patients with RCC were enrolled in the study. The retrospective study included patients with histopathologically verified RCC admitted to and nephrectomized at the Cancer Research Institute, Tomsk National Research Center, Russian Academy of Medical Sciences, Tomsk, Russian Federation. The patients underwent a physical examination, chest radiography, and computer tomography (CT) of the abdomen. Cavography or magnetic resonance imaging (MRI) was performed when vena cava tumor thrombus invasion was suspected. Patients with skeletal-associated pain or elevated serum alkaline phosphatase were assessed with bone scintigraphy. The patients were followed up according to a program, including regular clinical and radiological examinations. The median age of the patients was 57 years. The RCC therapy depends on the tumor size and its spreading to other parts of the body.

Localized RCC (T1-3N0M0) was diagnosed in 18 patients, metastatic RCC (T2-4N0-1M1) in 44 patients. All patients with localized RCC underwent surgery (partial nephrectomy or simple nephrectomy) and then followed up according to a program including regular clinical and radiological examinations. Patients with metastatic RCC received two cycles of preoperative targeted therapy with pazopanib at a dose of 800 mg daily for two months. Tumor response to targeted therapy was evaluated according to RECIST criteria. All patients underwent radical nephrectomy. The diagnosis was verified based on biopsy results.

The Local Committee approved the study for Medical Ethics, and all patients provided written informed consent (protocol code 4; 16 November 2018). Tumor tissue samples, histologically normal tissue samples adjacent to tumors, and metastatic tissues were used for investigation. Specimens were reviewed separately by two independent pathologists.

RNA extraction. The postoperative tumor samples were incubated in RNAlater solution (Ambion, 2130 Woodward St Ste 200, Austin (TX), 78744-1832, USA) for 24-h at +4 °C and then stored at −80 °C. Total RNA was extracted using the RNeasy Mini Kit (Qiagen, Bodenseeallee 20 78333 Stockach, Germany).

RT-qPCR. PCR was conducted in 25 μL reaction volumes containing 12.5 μL BioMaster HS-qPCR SYBR Blue (2X) (“Biolabmix”, 28, st. Ingenernaya, Novosibirsk, 630090, Russia) and 300 nanoM of each primers. *VHL:* F 5′-GGCAGGCGAATCTCTTGA-3′, R 5′-CTATTTCCTTTACTCAGCACCATT-3′; *PD-L2:* F 5′-GTTCCACATACCTCAAGTCCAA-3′, R 5′-ATAGCACTGTTCACTTCCCTCTT-3′; *PD-L1:* F 5′-AGGGAGAATGATGGATGTGAA-3′, R 5′-ATCATTCACAACCACACTCACAT-3′; *PD-1-1:* F 5′-CTGGGCGGTGCTACAACT-3′, R 5′-CTTCTGCCCTTCTCTCTGTCA-3′; *CAIX:* F 5′-GTTGCTGTCTCGCTTGGAA-3′, R 5′-CAGGGTGTCAGAGAGGGTGT-3′; *HIF-1′:* F 5′-CAAGAACCTACTGCTAATGCCA-3′, R 5′-TTTGGTGAGGCTGTCCGA-3′; *EPAS1:* F 5′-TGGAGTATGAAGAGCAAGCCT-3′, R 5′-GGGAACCTGCTCTTGCTGT-3′; *NFKB1:* F 5′-CGTGTAAACCAAAGCCCTAAA-3′, R 5′-AACCAAGAAAGGAAGCCAAGT-3′; *RELA:* F 5′-GGAGCACAGATACCACCAAGA-3′, R 5′-GGGTTGTTGTTGGTCTGGAT-3′; *VEGFA:* F 5′-AGGGCAGAATCATCACGAA-3′, R 5′-TCTTGCTCTATCTTTCTTTGGTCT-3′; *KDR:* F 5′-AACACAGCAGGAATCAGTCA-3′, R 5′-GTGGTGTCTGTGTCATCGGA-3′; *4E-BP1:* F 5′-CAGCCCTTTCTCCCTCACT-3′, R 5′-TTCCCAAGCACATCAACCT-3′; *AKT1:* F 5′-CGAGGACGCCAAGGAGA-3′, R 5′-GTCATCTTGGTCAGGTGGTGT-3′; *C-RAF:* F 5′-TGGTGTGTCCTGCTCCCT-3′, R 5′-ACTGCCTGCTACCTTACTTCCT-3′; *GSK3b:* F 5′-AGACAAGGACGGCAGCAA-3′, R 5′-TGGAGTAGAAGAAATAACGCAAT-3′; *70S kinase alpha:* F 5′-CAGCACAGCAAATCCTCAGA-3′, R 5′-ACACATCTCCCTCTCCACCTT-3′; *m-TOR:* F 5′-CCAAAGGCAACAAGCGAT-3′, R 5′-TTCACCAAACCGTCTCCAA-3′; *PDK1:* F 5′-TCACCAGGACAGCCAATACA-3′, R 5′-CTCCTCGGTCACTCATCTTCA-3′; *GAPDH:* F 5′-GGAAGTCAGGTGGAGCGA-3′, R 5′-GCAACAATATCCACTTTACCAGA-3′. At 95 °C for 10 min, a pre-incubation was to activate the Hot Start DNA polymerase and denature DNA and was followed by 45 amplification cycles of 95 °C denaturations at 95 °C for 10 s, 60 °C annealings at 60 °C for 20 s (iCycler iQ™, BioRad, 1000 Alfred Nobel Drive Hercules, CA 94547, USA).

The fold changes were calculated by ΔΔCt method (the total ΔΔCt = fold of cancerous/normal tissue gene level), using normal tissue. A ratio of specific mRNA/GADPH (GADPH as a respective control) amplification was then calculated.

Determination of p-VHL level in tissues. Electrophoresis SDS-PAGE (Laemmli) was used. The protein was transferred to a 0.2-/xm pore-sized PVDF membrane (GE Healthcare, Pollards Wood, Nightingales Ln, Chalfont Saint Giles HP8 4SP, UK), either at 150 mA or 100 V for one h by using a Bio-Rad Mini Trans-Blot electrophoresis cell. The membrane was incubated in a 1:2500 dilution of monoclonal mouse anti-p-VHL (Ser68) (Affinity Biosciences, 2928 Burnet Ave. Apt #6. Cincinnati, OH 45219, USA) at 4 °C overnight.

PVDF samples were incubated in Amersham ECL Western blotting detection analysis system (Amersham, Pollards Wood Nightingales Lane, Chalfont St Giles HP84SP, UK). The results were standardized using the beta-actin expression in a sample and were expressed in percentages to the protein content in non-transformed tissues. The analysis of the results was carried out using the ChemiDocTMTouch Imaging System, and their density was assessed using the ImageLab computer program (BioRad, 1000 Alfred Nobel Drive Hercules, CA 94547, USA). The level of protein in normal non-altered tissue was indicated as 100%.

Statistical analysis. Statistical analysis was performed using SPSS 19.0 software. Data were expressed as median and ranges. Mann–Whitney test was used for comparing differences in mean values. Nonparametric one-way ANOVA on ranks was carried out to test whether samples originate from the same distribution, which is used to compare two or more independent samples of equal or different sample sizes (Kruskal–Wallis test). Nonparametric correlation analysis was performed, and the Spearmen coefficient was calculated.

## 3. Results

### 3.1. PD-1, PD-L1, PD-L2 Expression in ccRCC Oncogenesis

The study revealed a relationship between PD-1 and PD-L1 ligand expression, increasing tumor size (Table 1). The mRNA level of the PD receptor decreased 5.17 and 10.1 times in patients with tumor size in patients with stage T3N0M0 and T2N0M0 compared with T1N0M0. There was also a decrease in the level of PD-L2 mRNA during tumor growth.

The presence of the disseminated form of the disease led to a decrease in PD expression by 4.75 times and an increase in PD-L2 expression by 5.3 times, respectively, compared with patients without distant metastases. A 2.69-fold decrease in the PD-L2 mRNA level in metastases was recorded compared to the primary tumor tissue.

The AKT/mTOR signaling cascade activation was found in the primary kidney tumor, both localized and disseminated cancers. PTEN loss results in the downstream activation of AKT/mTOR signaling in secondary cancer lesions and determines the survival of the overall ccRCC patients [19,20].

**Figure 1:**
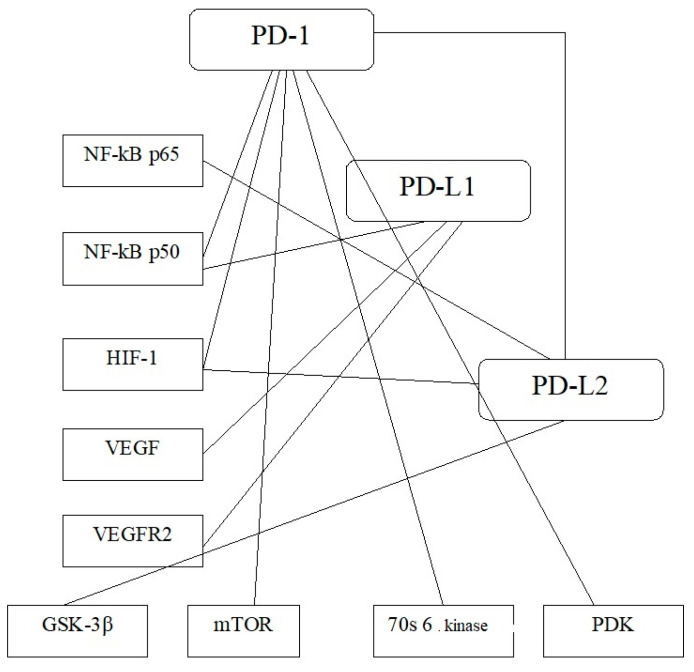
Association between the PD-1, PD-L1, PD-L2 with AKT/mTOR signaling pathway components, transcriptional and growth factors. Note: the correlation analysis revealed the interconnection between the PD receptors and their ligands in ccRCC. The found data indicates the complex associations between the molecular factors that trigger the immunity change.

The associations (Figure 1) were revealed between the expression of the PD-1 gene was associated with the expression of NF-κB p50 (r = 0.30; *p* < 0.05), HIF-1 (r = 0.30; *p* < 0.05), 70S 6 kinase (r = 0.30; *p* < 0.05), mTOR (r = 0.30; *p* < 0.05), PDK (r = 0.50; *p* < 0.05). The PD-L1 mRNA level correlated with NF-κB p50 (r = 0.30; *p* < 0.05), VEGFR2 (r = 0.30; *p* < 0.05), VEGF (r = 0.37; *p* < 0.05). In turn, the expression of the PD-L2 gene was associated with the mRNA level of NF-κB p65 (r = 0.41; *p* < 0.05), HIF-1 (r = 0.30; *p* < 0.05), GSK-3β 70s 6 kinase (r = 0.30; *p* < 0.05). In turn, associations were noted between PD and the PD-L2 ligand (r = 0.30; *p* < 0.05). However, there are no correlations between the VHL expression and p-VHL with studied indicators.

### 3.2. VHL Expression and p-VHL Content in ccRCC Oncogenesis

**Figure 2:**
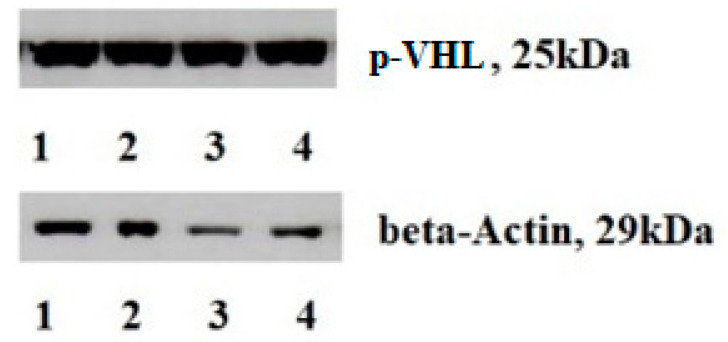
p-VHL content in ccRCC tissues and adjacent non-transformed ones. Note: 1, 3—cancers, 2, 4—non-transformed tissues; p-VHL content is a key oncogenic event in ccRCC development. It promotes and triggers the main biological processes resulting in metabolism modification, immune regulation, apoptosis, and cell movement.

The expression and content of VHL depended on the stage of the disease (criterion T) and the extent of the tumor process in ccRCC (Table 2). The VHL expression was increased by 12.5 times in patients with a T_3-4_N_0-1_M_1_ stage compared to T_1-2_N_0_M_0_. The found change was accompanied by a 5-fold decrease in the p-VHL content (Figure 2). The most significant changes were revealed when studying the prevalence of the tumor process. Substantial changes in the expression and protein level under study were observed in the metastatic tissue compared with the primary tumor. There was a decrease in the VHL expression in the metastases by 1.89 times and its content by 3.17 times compared with the primary tumor.

The expression and content of VHL depended on the stage of the disease (criterion T) and the extent of the tumor process in ccRCC (Table 3). The VHL expression was increased by 12.5 times in patients with a T_3-4_N_0-1_M_1_ stage than T_1-2_N_0_M_0_. The change was accompanied by a 5-fold decrease in the p-VHL in the patients with advanced cancers. The most significant changes were revealed when studying the prevalence of the tumor process. The expression and content of VHL are associated with the development of distant metastases. An increase in the VHL mRNA and its protein was recorded in patients with disseminated form by 32.0 and 2.68 times, respectively, of the disease, compared with patients with localized cancer.

### 3.3. AKT/mTOR Activation and Implementation of Transcriptional and Growth Factors in ccRCC Oncogenesis

A 2.8 times increase in mRNA level of HIF-2, NF-κB p50, respectively, was revealed in a primary tumor with stage T_3-4_N_0-1_M_1_ compared to T_1-2_N_0_M_0_. The reduction in CAIX mRNA level by 1.3 times was also found in these cancers. The distant metastases formation was accompanied by the increased nuclear factors NF-κBp50 and NF-κBp65 expression by 4.0 and 2.8 times, respectively, compared with patients with a localized form of the disease (Table 3). However, results in molecular markers in the transformed tissues in row primary localized, primary metastatic cancers and metastases show transcriptional factor NF-κB p50, HIF-1, CAIX, and VEGF overexpression.

Transcriptional and growth factors overexpression is the inducer of the molecular cascades and the most significant one—the AKT/mTOR signaling pathway. It is found the loss of PTEN mRNA in T_3-4_N_0-1_M_0-1_ in 3.23 times compared to the patients with T_1-2_N_0_M_0_ stage. The most pronounced changes were found in the metastatic kidneys and metastases. An 8.0 growth in PDK expression was detected in primary metastatic tissue compared to the localized one. The decreased mRNA level of c-RAF was accompanied by the cancer progression. A 1.76 times reduction in mRNA level was obtained in T_3-4_N_0-1_M_0-1_ stage patients compared to the patients with T_1-2_N_0_M_0_ stage. Additionally, the fall of gene expression indicated in primary metastatic cancers and affected distant organs. The exact change in mTOR expression (an 11.88 fall in gene expression) was also revealed in ccRCC, depending on the tumor size.

Thus, we indicate the transcriptional and growth factors induction, accompanied by the PD-L2 induction in ccRCC.

## 4. Discussion

The significance of immune system regulation has been verified in correlation analysis. The prevalence and distribution of PD-L2 are found to be correlated significantly with PD-L1 [5]. Previous studies have shown a relationship between the PD-L1 expression with a ccRCC poor prognosis [5,6,7,8,9]. The study revealed high levels of PD-L2 mRNA in patients with metastatic ccRCC. PD-L2 expression was observed in all tumor types and present in the stroma, tumor, and endothelial cells. However, PD-L2 was detected in the absence of PD-L1 in some tumor types [13]. The low rates in PD-1, PD-1, and PD-L2 were found in the tissue of metastases. The revealed data substantiate the contribution of heterogeneity and biological characteristics to the progression of the disease [11].

Multiple correlations between the PD-1, PD-L1, PD-L2, and transcriptional and growth factors are weak. However, taking into account their impact on oncogenesis, we suggest the summarized effect of molecular markers on the immune response modification.

We found the inner mechanism of immunity in ccRCC. The HIF-1, NF-κB p50, VEGF, CAIX overexpression trigger the immune response modulation, the key event in cancer progression and resistance induction.

VHL influences the progression of ccRCC tumors. ccRCC accounts for 2–3% of all tumors, the most frequent solid lesion in the kidney. The higher expression of VHL was correlated with the better disease-free survival in ccRCC patients using The Cancer Genome Atlas (TCGA) datasets [3].

Increased tumor immunogenicity during tumor progression is the foremost progression step associated with VHL inactivation [8,9,12,13,19]. Previous studies have revealed a relationship between VHL expression, the AKT/mTOR signaling cascade components, and transcriptional and growth factors [20,21,22,23], which possibly determines the response to targeted therapy [17,18,24,25,26]. In addition, the increase in gene expression and the content of transcriptional and growth factors have been shown in ccRCC. The expression and content of VHL depended on the stage of the disease (criterion T) and the extent of the tumor process in ccRCC.

## 5. Conclusions

The immune response is pivotal in oncogenesis in ccRCC. Growth in PD-L2 expression is associated with cancer progression. We found the prevalence of VHL expression in increased tumor growth and metastases development. The VHL expression and p-VHL content determine the aggressive cancer behavior and are elevated in disseminated tumors.

VHL mutation in kidney cancers is used to be a modulator of oncogenesis. Direct and indirect relationships in molecular factors determine the biological behavior and therapeutic effect. This work shows a novel mechanism for VHL tumor progression. It presents a new instrument and factor for targeting tumor-related pathologies with p-VHL/HIF altered function resulting in transcriptional, growth factors overexpression, and cancer immunity changes. We found the primary regulator among the programmed cell death receptors and their ligands. PD-L2 is revealed to be the potential marker of carcinogenesis and target anti-cancer therapy. This new insight in ccRCC progression may offer prominent opportunities for therapeutic intervention.

## Figures and Tables

**Table 1:** PD, PD-L1, PD-L2 expression in ccRCC tissues depending on tumor size and disease spreading, Me (Q1; Q3).

Indicator	Tumor Size			Cancer Dissemination		
	T_1_N_0_M_0_	T_2_N_0_M_0-1_	T_3_N_0_M_1_	Localized Cancers	Disseminated Cancers	Metastases
PD-1	4.19 (3.45; 20.40)	0.81 (0.11; 2.00)	0.41 (0.20; 1.94)	2.91 (1.44; 7.63)	0.61 (0.12; 1.94) *	0.57 (0.135; 1.82)
	Kruskal–Wallis Test: H = 11.95785 *p* = 0.0025					
PD-L1	2.85 (0.40; 6.77)	1.00 (0.19; 1.00)	0.32 (0.28; 0.50)	1.00 (0.25; 4.27)	0.37 (0.24; 1.00)	0.75 (0.375; 1.50)
	Kruskal–Wallis Test: H = 3.792281 *p* = 0.1501					
PD-L2	11.8 (1.49; 21.10)	1.14 (0.64; 2.00)	1.77 (0.50; 10.54)	1.28 (0.50; 2.17)	6.81 (2.16; 17.8) *	0.605 (0.435; 0.755) **
	Kruskal–Wallis Test: H = 4.837951 *p* = 0.0490					

Note: *—the significance of the differences with the localized cancers, *p* < 0.05; **—the significance of the differences compared to the primary metastatic tumor, *p* < 0.05.

**Table 2:** Expression and content of VHL in tumor tissue of patients depending on tumor size and disease spreading, Me (Q1; Q3).

Indicator	Tumor Size		Cancer Dissemination		
	T_1-2_N_0_M_0_	T_3-4_N_0-1_M_0-1_	Localized Cancer (T_1-2_N_0_M_0_)	Metastatic Cancers (T_1-4_N_0-1_M_1_)	Metastases
VHL expression, Relative Units	7.00 (2.00; 31.32)	87.50 (4.22; 128.00) *	2.00 (0.50;4.00)	64.00 (8.46; 128.00) *	1.09 (0.09; 5.44) **
VHL content,% to unaltered tissue	77.20 (42.25; 99.79)	15.30 (11.30; 46.07) *	55.00 (20.10; 93.61)	147.54 (100.54; 156.78) *	20.80 (10.60; 58.80) **

Note: *—the significance of the differences with the localized form, *p* < 0.05; **—the significance of the differences compared to the primary metastatic tumor, *p* < 0.05.

**Table 3:** Expression of nuclear factors HIF-1, HIF-1, NF-κBp50 and NF-κBp65, growth factors and components of AKT/m-TOR signaling pathway in tumor tissue of patients depending on tumor size and disease prevalence, Me (Q1; Q3).

Indicator	Tumor Size		Cancer Dissemination		
	T_1-2_N_0_M_0_	T_3-4_N_0-1_M_0-1_	Localized Cancer (T_1-2_N_0_M_0_)	Metastatic Cancers (T_1-4_N_0-1_M_1_)	Metastases
Transcriptional and growth factors					
HIF-1	1.29 (0.11; 2.53)	0.79 (0.09; 3.50)	1.20 (0.11; 3.30)	1.07 (0.01; 6.60)	20.80 (9.60; 35.30) **
			Kruskal–Wallis Test: H = 3.845614; *p* = 0.0156		
HIF-2	0.94 (0.13; 2.87)	2.68 (0.13; 128.00) *	1.00 (0.13; 4.00)	1.13 (0.01; 3.50)	2.95 (1.24; 12.27)
			Kruskal–Wallis Test: H = 3.792281; *p* = 0.1501		
NF-κB p50	1.07 (0.1; 12.72)	3.00 (0.03; 9.13) *	1.00 (0.08; 12.72)	4.08 (0.10; 6.92) **	6.00 (2.05; 28.14) **
			Kruskal–Wallis Test: H = 5.845614 df = 1 *p* = 0.0156		
NF-κB p65	1.39 (0.22; 4.60)	1.64 (0.66; 12.73)	1.00 (0.08; 12.72)	2.83 (0.01; 6.60) **	4.11 (0.14; 16.24)
			Kruskal–Wallis Test: H = 5.792281; *p* = 0.8501		
VEGF	1.39 (0.13; 25.00)	1.00 (0.13; 16.00)	1.35 (0.13; 25.00)	1.03 (0.00; 3.43)	21.75 (0.50; 31.31)
			Kruskal–Wallis Test: H = 5.792281; *p* = 0.0501		
CAIX	1.97 (0.31; 2.00)	2.56 (0.85; 5.00) *	0.81 (0.02; 2.00)	0.07 (0.01; 25.1)	1.85 (1.14; 2.28) **
			Kruskal–Wallis Test: H = 4.845614; *p* = 0.0056		
VEGFR2	0.74 (0.16; 8.57)	0.75 (0.25; 16.00)	1,00 (0.33; 16.00)	0.32 (0.04; 12.5)	0.91 (0.45; 1.82)
			Kruskal–Wallis Test: H = 5.792281; *p* = 0.8501		
AKT/mTOR signaling cascade components					
PTEN	4.72 (1.00; 8.78)	1.46 (0.50; 9.07) *	0.81 (0.50; 7.80)	2.23 (1.18; 8.11)	0.50 (0.00; 48.00)
			Kruskal–Wallis Test: H = 3.612530; *p* = 0.0490		
AKT	0.93 (0.63; 16.00)	1.48 (0.34; 3.42)	1.02 (0.54; 4.00)	4.57 (0.70; 23.23)	0.34 (0.28; 8.00)
			Kruskal–Wallis Test: H = 3.792281; *p* = 0.1501		
GSK-3β	3.78 (1.24; 17.00)	8.00 (4.00; 12.64)	2.59 (0.64; 9.50)	4.00 (0.54; 16.00)	0.24 (0.13; 8.00)
			Kruskal–Wallis Test: H = 3.792281; *p* = 0.1501		
PDK	3.65 (1.07; 15.60)	3.00 (0.51; 14.06)	5.48 (0.70; 17.30)	0.68 (0.03; 6.35) **	0.29 (0.01; 2.00)
			Kruskal–Wallis Test: H = 3.792281; *p* = 0.1501		
c-RAF	4.44 (1.96; 16.41)	2.51 (2.00; 8.00) *	1.90 (0.22; 4.88)	2.59 (0.16; 12.20)	0.25 (0.00; 10.00)
			Kruskal–Wallis Test: H = 4.371429 df = 1 *p* = 0.0365		
mTOR	4.28 (2.80; 16.00)	0.36 (0.16; 1.14) *	3.39 (0.84; 13.28)	1.15 (0.42; 7.47)	0.31 (0.25; 2.00)
			Kruskal–Wallis Test: H = 5.792281; *p* = 0.8501		
70S 6 kinase	2.01 (0.50; 5.58)	1.00 (0.20; 3.64)	0.98 (0.49; 4.64)	0.69 (0.55; 7.91)	0.13 (0.10; 1.18)
			Kruskal–Wallis Test: H = 4.792281; *p* = 0.1501		
4E-BP1	1.05 (0.35; 3.66)	1.00 (0.33; 2.00)	0.71 (0.28; 3.83)	0.83 (0.21; 6.22)	0.25 (0.00; 10.56)
			Kruskal–Wallis Test: H = 2.792281; *p* = 0.5501		

Note: *—the significance of the differences with the T_1-2_N_0_M_0_ stage, *p* < 0.05; **—the significance of the differences compared to the localized tumor, *p* < 0.05.
